# Induction of Peroxiredoxin 1 by Hypoxia Regulates Heme Oxygenase-1 via NF-κB in Oral Cancer

**DOI:** 10.1371/journal.pone.0105994

**Published:** 2014-08-27

**Authors:** Min Zhang, Min Hou, Lihua Ge, Congcong Miao, Jianfei Zhang, Xinying Jing, Ni Shi, Tong Chen, Xiaofei Tang

**Affiliations:** 1 Institute of Dental Research, Beijing Stomatological Hospital and School of Stomatology, Capital Medical University, Beijing, China; 2 Division of Medical Oncology, Department of Internal Medicine, The Arthur G. James Cancer Hospital and Richard J. Solove Research Institute, The Ohio State University, Columbus, Ohio, United States of America; The University of Hong Kong, China

## Abstract

Overexpression of peroxiredoxin 1 (Prx1) has been observed in numerous cancers including oral squamous cell carcinoma (OSCC). The precise molecular mechanism of up-regulation of Prx1 in carcinogenesis, however, is still poorly understood. The objective of this study is to investigate the relationship between Prx1 and hypoxia, and potential mechanism(s) of Prx1 in OSCC cell line SCC15 and xenograft model. We treated wild-type and Prx1 knockdown SCC15 cells with transient hypoxia followed by reoxygenation. We detected the condition of hypoxia, production of reactive oxygen species (ROS), and expression and/or activity of Prx1, heme oxygenase 1 (HO-1) and nuclear factor-kappa B (NF-κB). We found that hypoxia induces ROS accumulation, up-regulates Prx1, increases NF-κB translocation and DNA binding activity, and down-regulates HO-1 *in vitro*. In Prx1 knockdown cells, the expression level of HO-1 was increased, while NFκB translocation and DNA binding activity were decreased after hypoxia or hypoxia/reoxygenation treatment. Moreover, we mimicked the dynamic oxygenation tumor microenvironment in xenograft model and assessed the above indices in tumors with the maximal diameter of 2 mm, 5 mm, 10 mm or 15 mm, respectively. Our data showed that tumor hypoxic condition and expression of Prx1 are significantly associated with tumor growth. The expression of HO-1 and NF-κB, and NF-κB DNA binding activity were significantly elevated in 15 mm tumors, and the level of 8-hydroxydeoxyguanosine was increased in 10 mm and 15 mm tumors, compared to those in size of 2 mm. The results from this study provide experimental evidence that overexpression of Prx1 is associated with hypoxia, and Prx1/NF-κB/HO-1 signaling pathway may be involved in oral carcinogenesis.

## Introduction

Oral squamous cell carcinoma (OSCC) is the most common head and neck cancer worldwide. Despite of the advantages of surgery, chemotherapy and radiotherapy, the 5-year survival rate of OSCC has not improved markedly in the past 30 years [1]–[3]. Metastasis and chemotherapy/radiotherapy resistance are still main concerns in clinical cancer therapy. Hypoxia, a common feature of cancer, promotes tumor cell invasion and metastasis in tumor microenvironment leading to the resistance of tumor cells to chemotherapy and radiotherapy [4], [5]. It has been reported that the pathological features including active invasiveness, lymph node metastasis and vascular proliferation are associated with tumor tissue hypoxia in OSCC [6], [7]. Exposure to hypobaric hypoxia leads to a significant increase in production of reactive oxygen species (ROS) in animals. The accumulation of ROS induced by hypoxia can result in oxidative stress and tumor progression [8]. The ROS-mediated response can be regulated by antioxidants and antioxidant enzyme systems, such as superoxide dismutase, catalase and thioredoxin/peroxiredoxin [9].

Peroxiredoxins (Prxs) are a superfamily of multifunctional antioxidant thioredoxin-dependent peroxidases. Peroxiredoxin 1 (Prx1) is a major member in Prxs family and acts as an antioxidant to scavenge ROS in a wide range of organisms. Prx1 is involved in multiple biological conditions/activities including oxidative stress, cell proliferation and cell apoptosis [9]. Prx1 is overexpressed in many malignancies including esophagus, lung, breast and pancreatic cancers. Elevated Prx1 expression is associated with diminished overall survival and poor clinical outcome [10], [11]. Overexpression of Prx1 has also been reported in OSCC, but the precise molecular mechanism of Prx1 in oral carcinogenesis remains obscure [12]. The expression of nuclear factor-kappa B (NF-κB) and Heme oxygenease-1 (HO-1) are increased in OSCC [13], [14]. Numerous studies showed that NF-κB responses to hypoxia–reoxygenation *in vitro* [15]. NF-κB can be regulated by Prx1, through its initial activation in cytoplasm [16] or by altering the concentration of oxidant leading to the oxidative inactivation of NF-κB [17]. HO-1, a 32-kDa heat shock protein, can catalyze the oxidative degradation of heme to biliverdin, which is subsequently reduced to bilirubin. HO-1 can be induced by hypoxia, heat shock and cytotoxic oxidants [18], [19]. HO-1 is one of the targets of NF-κB under stressful conditions [20]–[23]. Although the mechanism of Prx1 in oxidant stress is not clear, multiple evidences suggest that Prx1 may response to hypoxia and regulate NF-κB pathway cascade. In this study, we investigated the alteration of Prx1 in response to hypoxia and evaluated the potential correlations between Prx1 and NF-κB/HO-1 using oral cancer cell system and xenograft model.

## Materials and Methods

### Cell culture

Human OSCC cells, SCC15, (ATCC, Manassas, VA) were maintained in DMEM-F12 supplemented with 10% (v/v) fetal bovine serum (FBS) (Gibco, USA) containing 100 units/mL penicillin and 100 µg/mL streptomycin. SCC15 cells were grown at 37°C in an atmosphere of 5% CO_2_ and 95% air. To knock down Prx1, SCC15 cells were transfected with Prx1 shRNA Plasmid (Santa Cruza Biotechnology) using Lipofectamine 2000 (Invitrogen, USA) according to the manufacturer’s instructions. Control shRNA Plasmid-A (Santa Cruza Biotechnology) was transfected as control. The efficiency of Prx1 shRNA knockdown was determined by qRT-PCR and western blot analyses.

### Pimonidazole staining in SCC15 cells

Cells were grown on coverslips for 24 h prior to hypoxia treatment. During hypoxia treatment, cells were put in a hypoxia incubator with gas containing 1% O_2_, 5% CO_2_ and 94% N_2_ at 37°C for different periods of time. 200 µM of pimonidazole was added to the medium and incubated for 30 min. After washed with PBS, cells were fixed with paraformaldehyde (3%) at room temperature for 10 min and subsequently washed again with PBS. After blocked with BSA, coverslips were incubated with hypoxyprobe-1 mouse monoclonal antibody MAB1 (1∶100) in the Hypoxyprobe-1 Plus kit (Chemicon Int., Temecula, CA) overnight. A secondary FITC-conjugated anti-mouse antibody was used. Coverslips were mounted and immunostained cells were examined on an Olympus IX71 microscope (Tokyo, Japan).

### ROS detection

The production of intracellular ROS in cells was measured with 2′,7′-dichlorodihydrofluorescein diacetate (DCF-DA) using FACS analysis. The SCC15 cells were collected and suspended in 500 µL diluted DCF-DA. The mixture was incubated at 37°C for 20 min and then washed twice with PBS. The samples were analyzed by flow cytometer within 1 h. Data was normalized to the values from controls. The mean DCF fluorescence intensity was measured with excitation at 488 nm and emission at 525 nm. The experiments were performed in triplicate.

### Immunofluorescence

Cells grown on coverslips were rinsed with PBS, fixed and permeabilized in acetone at −20°C for 10 min. After incubation with PBS containing 5% BSA for 1 h, monoclonal anti-NF-κB antibody (Cell Signaling Technology, 1∶100 in PBS-BSA) was added at 4°C and incubated overnight. Coverslips were washed with PBS, incubated with Alexa Fluor 546 anti-rabbit (Molecular Probes, 1∶500) secondary antibody at room temperature for 1 h, and mounted in Dako Cytomation fluorescent mounting medium. Confocal images were collected using a Leica SP2 laser scanning confocal microscope equipped with UV excitation, an argon laser, a 633/1.32 OIL PH3 CS objective, and a confocal pinhole set at 1 Airy unit. All the confocal images were single optical sections.

### Xenograft model

Forty male BALB/c nude mice (Beijing Vital River Laboratories, China) were used to produce tumorigenicity of the SCC15 cells *in vivo*. The experiment protocol was approved by the local ethical committee for animal use. The SCC15 cells (6×10^6^) were suspended in 100 µL of phosphate-buffered saline and were transplanted subcutaneously in the right and left flanks of 4-week old nude mice. The tumor growth was monitored every 3 days after transplantation. The tumors were harvested when the maximal diameter of tumors reached 2 mm, 5 mm, 10 mm or 15 mm, respectively. To label hypoxic cells, the nude mice were injected intraperitoneally with 0.1 mL saline containing pimonidazole hydrochloride (1-[(2-hydroxyl-3-piperidinyl) propyl)-2-nitroimidazole hydrochloride) at a dosage of 60 mg/kg body weight 1 hour before tumor excision. Mice were implemented euthanasia and the tumor specimens were removed and immediately stored in liquid nitrogen for molecular/cellular analysis, or in formalin to make paraffin-embedded tissue blocks. Total 80 tumors were divided into four groups according to the tumor diameter (20 tumors/size).

### Pimonidazole staining in tumors

The paraffin-embedded blocks with tumor samples were sectioned for pimonidazole staining. The sections were incubated with primary rabbit anti-pimonidazole antiserum hypoxyprobe-1 MAB1 (dilution: 1∶50; Chemicon, USA) at 4°C overnight. To evaluate the condition of hypoxia, five representative light microscopic areas were computed on each section (magnification ×200) and the mean optical density (MOD) was calculated using the Image Pro Plus 7.0 analyzer, MOD = IOD/area.

### qRT-PCR analysis

Total RNA was extracted from cultured cells and tumor tissues using TRIzol (Invitrogen Life Technologies, USA) according to the manufacturer’s instructions. cDNA was synthesized by reverse transcribing 2 µg RNA with the High-Capacity cDNA Reverse Transcription Kit (Applied Biosystems, USA). One-microliter aliquots of cDNA were used as templates. The FAMTM Dye/MGB probes of Prx1, HO-1 and ß-actin were synthesized by ABI (Assay ID: Prx1, HS0060202; HO-1, HS00015796; human ACTB, 4352935E). For data analysis, the 2^−ΔΔCT^ method was used with normalization of data of interested genes to housekeeping gene ß-actin. The experiments were conducted in triplicate.

### Western blot analysis

Cells and tumor tissues were lysed in immunoprecipitation assay buffer (50 mM Tris-Cl [pH 7.4], 1% NP40, 150 mM NaCl, 1 mM EDTA, 1 M phenylmethylsulfonyl fluoride, 10 µg each of aprotinin and leupeptin, and 1 mM Na3VO4). After centrifuged at 12,000 g for 30 min, the supernatant was collected and protein concentration was determined using the Lowry method. Equal amounts of protein were separated on 12% SDS-PAGE gels and blotted onto nitrocellulose membranes. The blots were incubated with anti-Prx1 (1∶5000, Upstate, USA) and anti-HO-1 (1∶2000, Abcam, USA) antibody. Antibody of β-actin (Sigma, St. Louis, MO) was used as a loading control. Immunoreactive bands were detected with horseradish peroxidase-conjugated secondary antibodies and enhanced by chemiluminescence reagents (Amersham Biosciences, Piscataway, NJ). The experiments were performed in triplicate.

### NF-κB DNA binding activity detection

The nuclear protein was extracted from cells and tumor tissues using NE-PER Nuclear and Cytoplasmic Extraction Reagents (Thermo, USA). In brief, cells and tissues were suspended in hypotonic buffer and incubated on ice for 10 min followed by centrifuged at 12,000 g and 4°C for 10 min to precipitate nuclei. After washed in the hypotonic buffer, the nuclei were lysed in a lysis buffer to get the nuclei extracts. NF-κB DNA binding activity was detected using the NF-κB (p65) Transcription Factor Assay Kit (Cayman Chemical Company, USA).

### Immunohistochemistry

The paraffin-embedded blocks were sectioned for immunohistochemistry analysis. Sections were treated with 20 mg/L proteinase K (dilution: 1∶1000; Sigma-Aldrich, USA) at 37°C for 30 min for antigen retrieval. After blocking the endogenous peroxidase activity with 0.3% hydrogen peroxidase for 15 min, the sections were treated with 8-hydroxydeoxyguanosine (8-OHdG; 1∶8000; Abcam, USA) or NF-κB (Cell Signaling Technology, 1∶100) primary antibody at 4°C overnight. The slides were incubated in biotinylated secondary IgG antibodies at 37°C for 30 min, and then visualized using DAB for 2–5 min. Mayer’s hematoxylin was used to counterstain the sections, which were then dehydrated and mounted. For negative control, PBS was used in place of primary antibodies. The cells with positive staining were determined by counting the percentage of stained cells using the Image Pro Plus 7.0 analyzer. A minimum of 1,000 cells were counted for each tumor specimen.

### Statistical analysis

The expression levels of Prx1, HO-1, NF-κB and 8-OHdG, and data from ROS production, pimonidazole staining, immunofluorescence and NF-κB DNA binding activity were analyzed and compared using one-way analysis of variance (ANOVA). All statistical analysis was carried out using SPSS Software for Windows 17.0. Differences were considered statistically significant at *P*<0.05. All *P* values were two-sided.

## Results

### Prx1 knockdown increases hypoxia and intracellular ROS production

**Figure 1:**
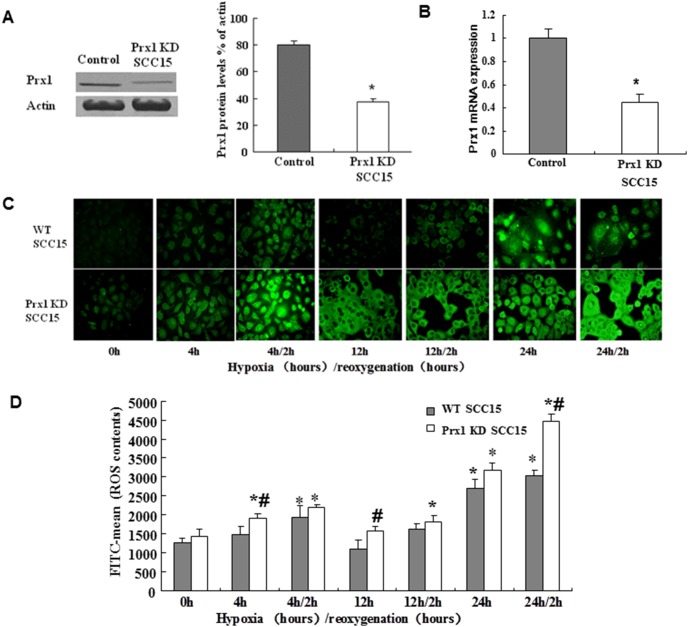
Prx1 knockdown increases hypoxia and intracellular ROS production in SCC15 cells. A, Prx1 protein was significantly decreased by shRNA transfection. B, Prx1 mRNA was significantly reduced by shRNA transfection. C, Pimonidazole staining showed the increased hypoxia status in cells after hypoxia/reoxygenation treatment. D, Intercellular ROS level identified by flow cytometry in cells after hypoxia/reoxygenation treatment. *P<0.05 vs. WT SCC15 cells; ^#^ *P*<0.05 vs. control hypoxic WT SCC15 cells.

We used shRNA plasmid to knock down Prx1 in SCC15 cells. As shown in Figure 1A and 1B, the levels of Prx1 mRNA and protein expression were reduced to 40–50% by shRNA transfection in SCC15 cells. After Prx1 knockdown, we detected the hypoxic condition in both Prx1 knockdown cells and control cells, which were transfected with empty vector. Our data showed that hypoxic condition was increased by hypoxia or hypoxia/reoxygenation except 12-h hypoxia treatment in control cells (Figure 1C, upper panel). In Prx1 knockdown cells, the hypoxic condition increased more significantly compared to control cells (Figure 1C, lower pannel). The highest hypoxia was observed in Prx1 knockdown cells after 24 h hypoxia/2 h reoxygenation treatment, and 2 h reoxygenation treatment did not decrease the hypoxia level elevated by hypoxia treatment. We also detected the production of intracellular ROS. Similar to hypoxia condition, the production of intracellular ROS was increased by hypoxia treatment except 12 h hypoxia and 12 h hypoxia/2 h reoxygenation treatment (Figure 1D). The accumulation of intracellular ROS is higher in Prx1 knockdown cells compared to it in control cells.

### Prx1 negatively regulates HO-1 under hypoxic conditions

**Figure 2:**
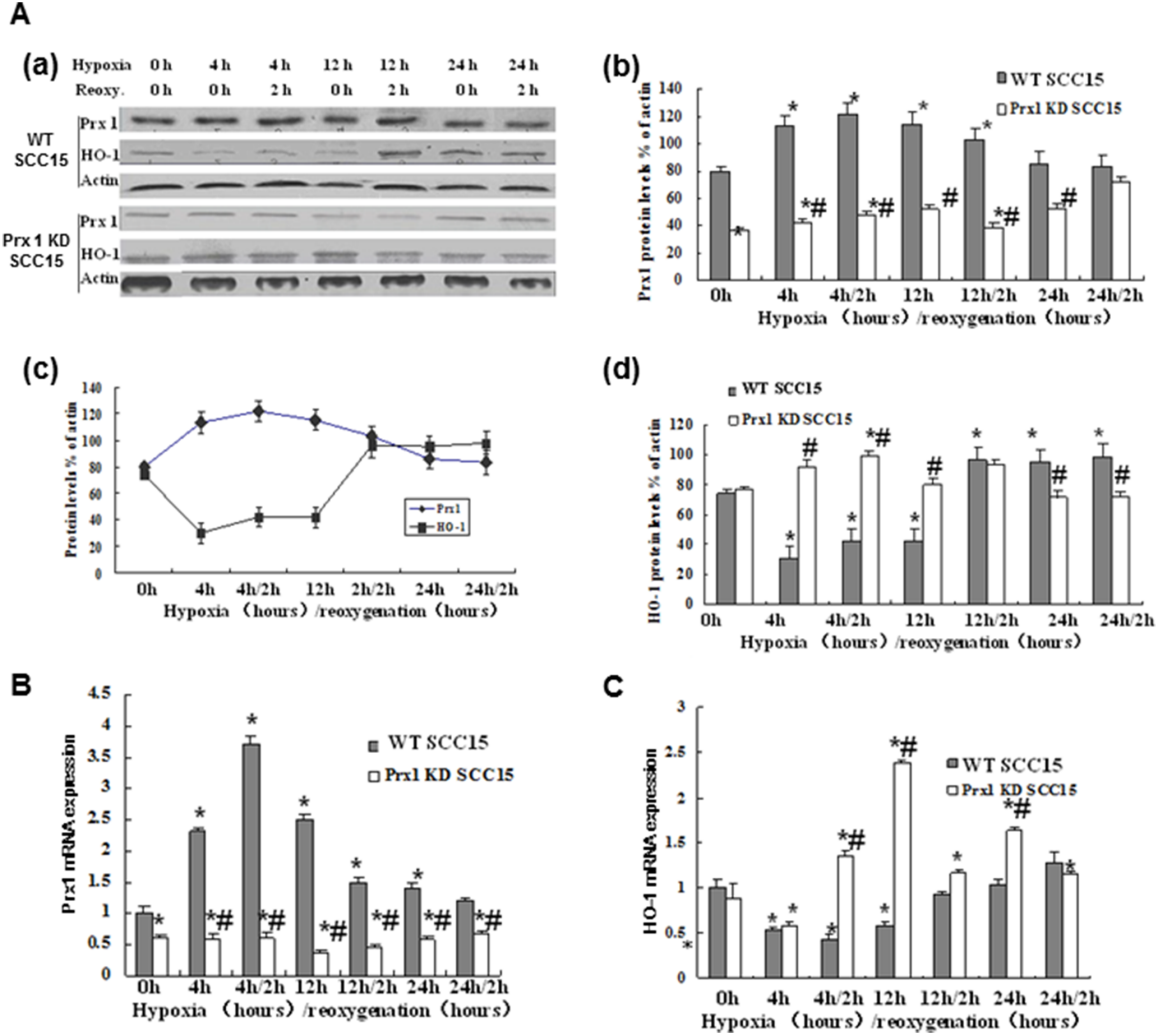
Expression of Prx1 and HO-1 in SCC15 cells. A (a), protein expression of Prx-1 and HO-1 detected by Western blot after hypoxia/reoxygenation treatment; A (b and d), Prx1 and HO-1 protein quantitation relative to β-actin; A (c), correlation of Prx-1 and HO-1 after hypoxia/reoxygenation treatment. B and C, mRNA expression of Prx-1 and HO-1 detected by qRT-PCR after hypoxia/reoxygenation treatment. *P<0.05 vs. WT SCC15 cells; ^#^ *P*<0.05 vs. control hypoxic WT SCC15 cells.

In order to determine whether Prx1 was related to HO-1 in SCC15 cells under hypoxic conditions, we assessed the expression levels of Prx1 and HO-1 after hypoxic treatment. As shown in Figure 2A (a, b), the protein expression of Prx1 was increased by hypoxic treatment. However, the protein expression of HO-1 was decreased at initial stages of hypoxia when Prx1 expression was increased, and then increased while Prx1 expression decreased in 12 h hypoxia/2 h reoxygenation, 24 h hypoxia and 24 h hypoxia/2 h reoxygenation, as indicated in Figure 2A (c and d). In 4 h hypoxia/2 h reoxygenation group, the level of Prx1 protein was increased by 50% while HO-1 protein was down-regulated by 30% compared with untreated cells (Figure 2A–b and d). However, in Prx1 knockdown cells, HO-1 was significantly up-regulated after hypoxia or hypoxia/reoxygenantin treatment except for the 24 h group. The protein expression of HO-1 was increased by 30% (Figure 2A–d), and mRNA level was increased by 1.4-fold in Prx1 knockdown SCC15 cells treated with 4 h hypoxia/2 h reoxygenation compared with untreated cells (Figure 2C). The mRNA expression of Prx1 was increased by 2.7-fold while the expression of HO-1 mRNA was down-regulated by 40% (Figure 2B and 2C).

### Prx1 knockdown inhibits NF-κB translocation and DNA binding activity

**Figure 3:**
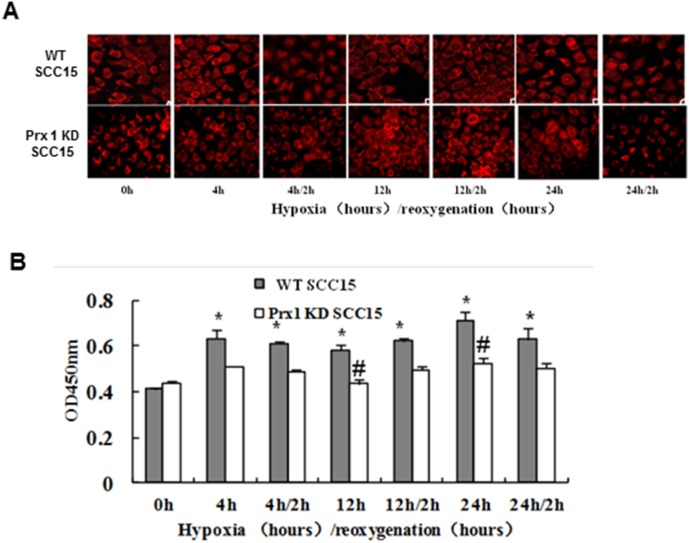
NF-κB DNA binding activity in SCC15 cells. A, Endogenic nuclei expression of NF-κB. B, NF-κB DNA binding activity detected by ELISA. *P<0.05 vs. WT SCC15 cells; ^#^ *P*<0.05 vs. control hypoxic WT SCC15 cells.

We detected the endogenic NF-κB expression in cells after hypoxia treatment. We observed an increased nucleus expression of NF-κB in SCC15 cells after treatment, especially in 24 h hypoxia group (Figure 3A, upper panel). Moreover, in Prx1 knockdown cells, NF-κB translocation from cytoplasm to nucleus was decreased compared with control cells (Figure 3A, lower panel). We then detected NF-κB activity in cells after hypoxia treatment. We found that NF-κB p65 DNA binding ability was significantly decreased in Prx1 knockdown cells after hypoxia treatment compared to control cells (Figure 3B).

### Hypoxia is associated with tumor growth in xenograft model

**Figure 4:**
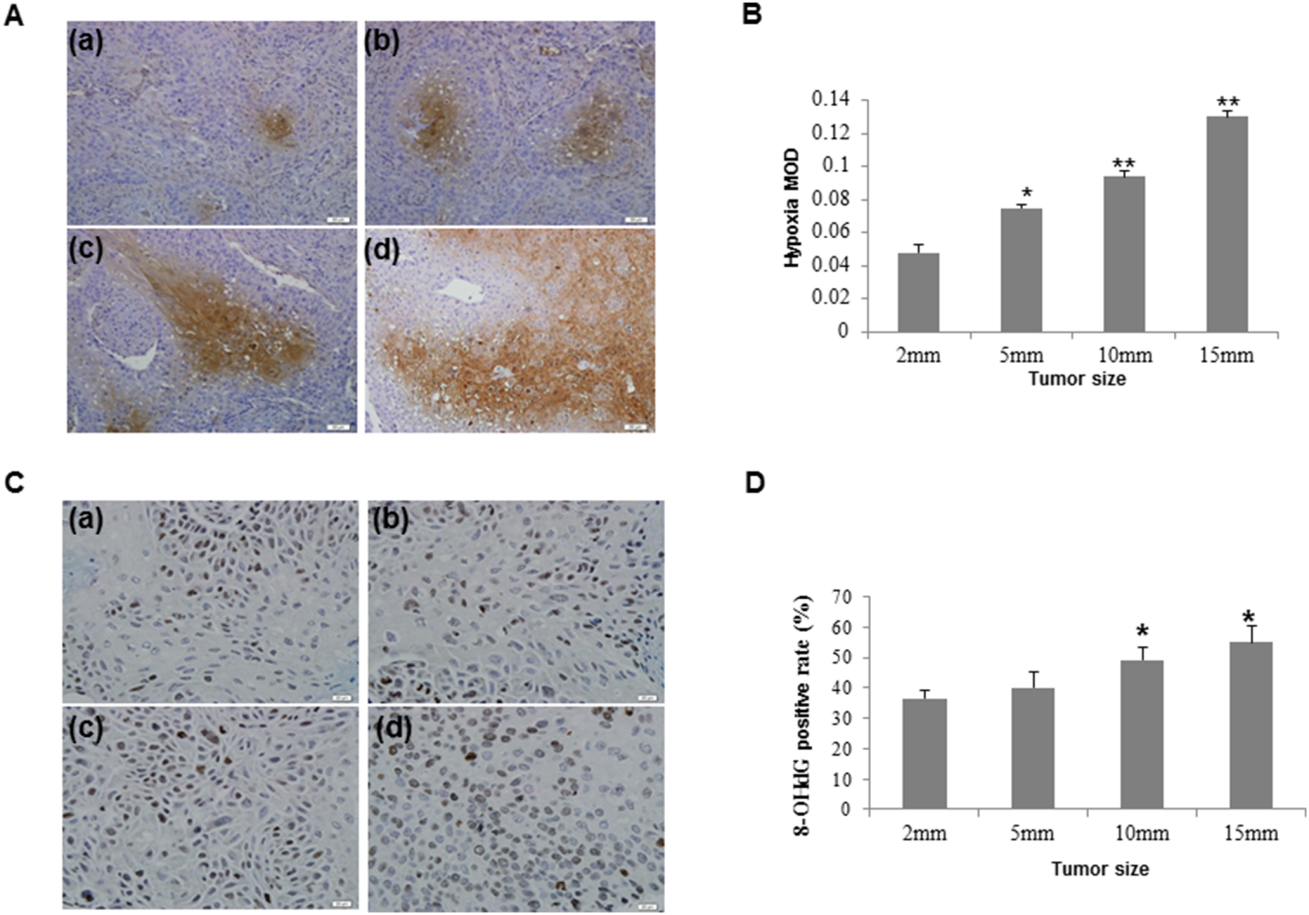
Pimonidazole staining and 8-OHdG expression in xenograft tumors. A, Hypoxia was detected in 2 mm (a), 5 mm (b), 10 mm (c) and 15 mm (d) tumors by pimonidazole staining. B, Hypoxia MOD significantly increases in 5 mm, 10 mm and 15 mm tumors compared to 2 mm tumors. C, 8-OHdG was detected by immunohistochemistry in 2 mm (a), 5 mm (b), 10 mm (c) and 15 mm (d) tumors. D, The level of 8-OHdG was higher in 10 mm and 15 mm tumors compared to 2 mm tumors. *P<0.05; **P<0.01.

To evaluate the change of the hypoxic conditions during the tumor development, we assessed hypoxia in tumors with maximal diameter of 2 mm, 5 mm, 10 mm or 15 mm. As shown in Figure 4A, the small and scattered positive cells were observed in 2 mm tumors. In 5 mm, 10 mm and 15 mm tumors, the hypoxic cells were mainly located at the centers of the cancer nests. The hypoxia in 5 mm, 10 mm and 15 mm tumors was significantly higher than it in 2 mm tumors (Figure 4B). We also detected the level of 8-OHdG to assess the oxidative DNA injury in tumor cells. The expression of 8-OHdG increased from 2 mm to 15 mm tumors (Figure 4C). It was significantly higher in 10 mm and 15 mm tumors compared to 2 mm tumors (Figure 4D).

### Overexpression of Prx1 and HO-1 during tumorigenesis in xenograft model

**Figure 5:**
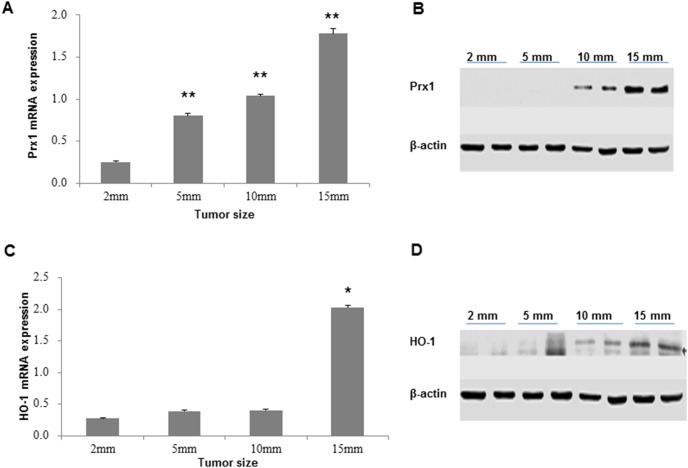
Expression of Prx1 and HO-1 in xenograft tumors. A and C, mRNA expression of Prx1 and HO-1 detected by qRT-PCR. B and D, Protein expression of Prx1 and HO-1 detected by western blot. *P<0.05; **P<0.01.

As shown in Figure 5A, the mRNA expression of Prx1 significantly increased in 5 mm, 10 mm and 15 mm tumors compared to 2 mm tumors. The protein expression of Prx1 was also elevated in 10 mm and 15 mm tumors (Figure 5B). Overexpression of HO-1 was observed in 15 mm tumors as indicated in Figure 5C and 5D.

### NF-κB translocation and DNA binding activity are increased in xenograft model

**Figure 6:**
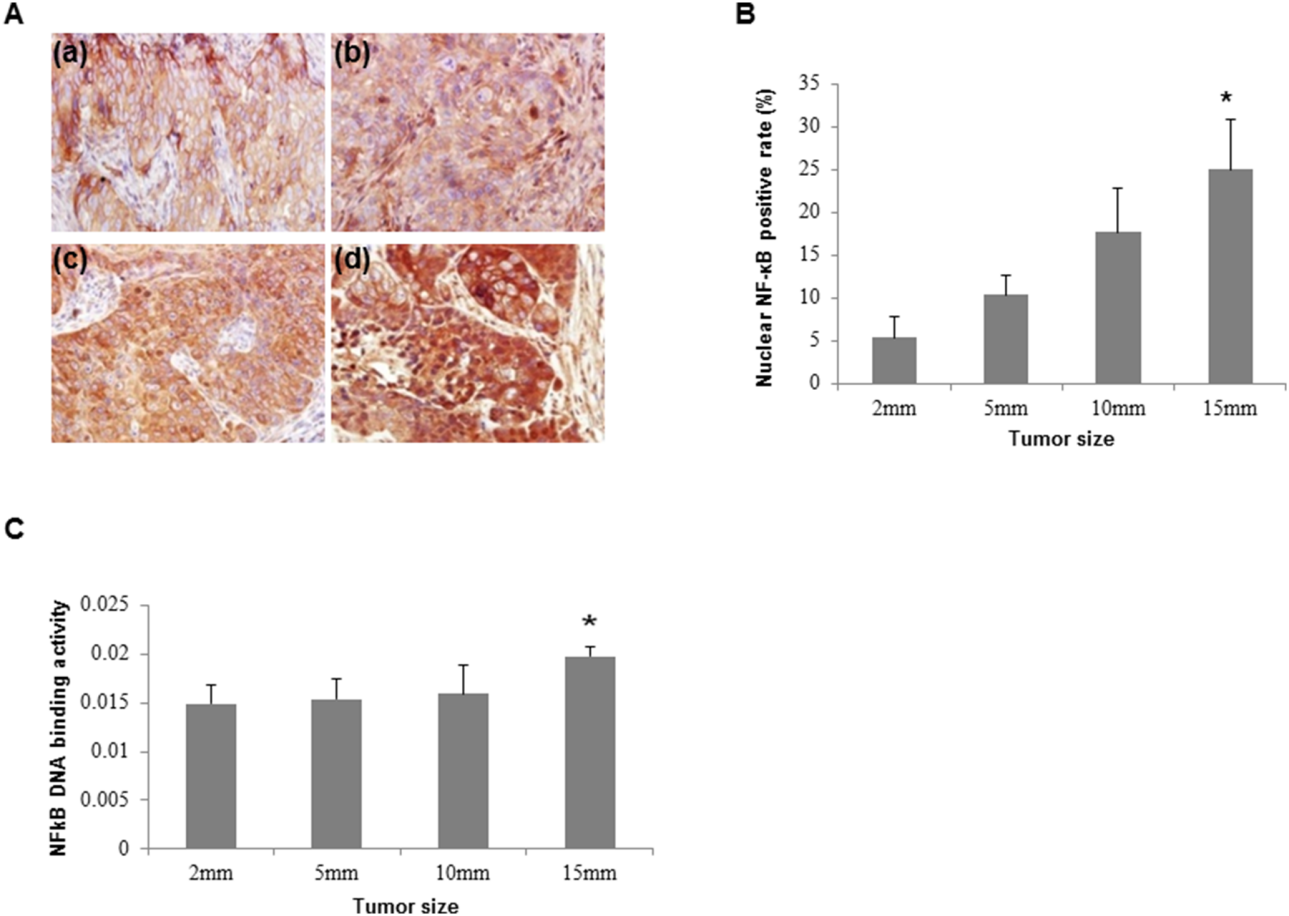
NF-κB expression and DNA binding activity in xenograft tumors. A, NF-κB expression was detected by immunohistochemistry in 2 mm (a), 5 mm (b), 10 mm (c) and 15 mm (d) tumors. B, The percentage of nuclear NF-κB positive cells is significantly increased in 15 mm tumors compared to it in 2 mm tumors. C, NF-κB DNA binding activity was significantly higher in 15 mm tumors compared to 2 mm tumors. *P<0.05; **P<0.01.

NF-κB translocation and DNA binding activity both were elevated when tumor developed. The immunohistochemistry analysis showed that the expression of NF-κB was increased significantly in 15 mm tumors compared to 2 mm tumors (Figure 6A and 6B). Similarly, NF-κB DNA binding activity was significantly elevated in 15 mm tumors than 2 mm tumors (Figure 6C).

## Discussion

The overall objective of this study is to explore the role of Prx1 in hypoxia in OSCC. First, we used shRNA to knock down Prx1 in SCC15 cells and then detected Prx1, NF-κB and HO-1 in hypoxia. We also investigated the Prx1 with hypoxia during tumor development in xenograft model. Our data showed that after exposure to hypoxia or hypoxia/reoxygenation, intracellular hypoxia and ROS levels were aggravated. We also found that HO-1 expression was up-regulated in Prx1 knockdown cells, whereas NF-κB translocation and DNA binding activity were decreased. Taken together, these data suggest that the elevated accumulation of ROS induced by hypoxia can up-regulate Prx1, which can activates NF-κB and negatively regulates HO-1 in hypoxia-induced oral cancer cells. Our *in vivo* data showed that overexpression of Prx1 is associated with hypoxia and tumor growth. These results suggest that Prx1/NF-κB/HO-1 signaling pathway may play a key role in carcinogenesis of hypoxia-induced oral cancer.

One of the major functions of Prx1 is thioredoxin-dependent peroxidase activity relying exclusively on the cysteine at the N-terminal region [24]. It scavenges extra ROS as an antioxidant. Hypoxia increases intercellular ROS levels via the mitochondrial electron transport chain. Hypoxia is one of the key factors influencing tumor initiation and progression through increasing oxidative DNA damage [25]. In recent years, the overexpression of Prx1 was reported to play an important role in many malignancies due to its critical role in pathogenesis of hypoxia. In lung cancer cells, hypoxia/reoxygenation can increase Prx1 expression by activating nuclear factor erythroid 2–related factor 2 (Nrf2), an important transcription factor involved in oxidant stress [26]. The loss of Prx1 expression can enhance the sensitivity to oxidants and therefore, increase ROS production and oxidative DNA damage [27]. In the present study, the mRNA and protein expression of Prx1 was up-regulated in SCC15 cells by either hypoxia (4 h, 12 h) alone or followed by reoxygenation (2 h). In another study reported by Kim *et al.,* Prx1 was only up-regulated by hypoxia/reoxygenation, but not by hypoxia alone [27]. We found that the SCC15 cells are still hypoxic after 2 h reoxygenation treatment, which indicates that Prx1 could still be induced by hypoxia, even after 2 h reoxygenation treatment. Our result suggests that hypoxia is closely related to the overexpression of Prx1 in OSCC. The up-regulation of Prx1 increases the cells’ ability to remove extra ROS and protect the tumor cells, which may enhance tumor progression and reduce the efficacies of chemo- and radiotherapies.

A recent study showed that in Hela cells, cytoplasmic Prx1 altered cytoplasmic NF-κB translocation into the nucleus. Nuclear Prx1 regulates NF-κB/DNA binding through elimination of H_2_O_2_ [23]. Wang *et al.* reported that Prx1 interacts with NF-κB at the DNA level and presumably modulates its transcriptional activity in breast cancer cells [10]. As a potent antioxidant, HO-1 facilitates tumor progression in a tissue specific manner. Studies have indicated that NF-κB induces expression of HO-1 in tumor tissues [28]–[31]. Moreover, numerous studies suggested a direct relationship between NF-κB and HO-1, but the function of NF-κB in regulating HO-1 expression in human cells is controversial [32]–[35]. In this study, our data showed that hypoxia induces Prx1 and NF-κB, and Prx1 regulates HO-1 via activating NF-κB.

Prx1 has been recently identified as an endogenous ligand for toll-like receptor (TLR) ligands [36]. In normoxic conditions, Prx1 can enhance expression of vascular endothelial growth factor (VEGF) via induction of hypoxia-inducible factor 1-alpha promoter activity through Prx1: TLR4 interaction. This process is mediated by NF-κB. NF-κB interacts with the VEGF promoter, however, is not required for Prx1, which suggests that NF-κB can regulate VEGF without Prx1 induction [37]. Prx1 and HO-1 are both characterized as oxidative stress-inducible and heme-related proteins. Prx1 has heme-binding activity. The thiol-specific antioxidant activity of Prx1 can be inhibited by heme. Co-expression or co-induction of Prx1 and HO-1 has been observed in some tissues or cells, indicating that Prx1 and HO-1 proteins may co-localize and interact to exhibit antioxidant activities [15]. Studies have shown that the expression of HO-1 is mainly up-regulated by Nrf2. Nrf2 is also one of the key transcription factors for Prx1 gene expression in hypoxic cancer cells. For the first time, we report that Prx1 and HO-1 could have a negative regulatory relationship in an indirect manner. The precise molecular mechanisms, however, needs to be confirmed and investigated in the future study.

In OSCC xenograft model, we found similar results that significant elevation of HO-1 lags behind that of Prx1. Under hypoxic condition and oxidative DNA damage, Prx1 and HO-1 are upregulated and NF-κB DNA binding activity is enhanced. Our data suggests that Prx1 plays antioxidative role by activating NF-κB and regulating HO-1 in hypoxia-related OSCC progression. In conclusion, we found that hypoxia plays an important role in OSCC through regulating Prx1/NF-κB/HO-1 signaling pathway. Our study provides a systemic examination of Prx1 in cell systems and xenograft model of OSCC, each having specific advantages for biomarker research. Further studies on functional role of Prx1 in oral carcinogenesis are warranted. Information from this study may be helpful in developing chemopreventive/therapeutic agents targeting Prx1.

## References

[1] Warnakulasuriya S (2009) Global epidemiology of oral and oropharyngeal cancer. Oral Oncol 45(4–5): 309–16. 18804401 10.1016/j.oraloncology.2008.06.002

[2] Warnakulasuriya S (2009) Causes of oral cancer–an appraisal of controversies. Br Dent J 207: 471–475. 19946320 10.1038/sj.bdj.2009.1009

[3] Bagan JV, Scully C (2008) Recent advances in Oral Oncology 2007: epidemiology, aetiopathogenesis, diagnosis and prognostication. Oral Oncol 44: 103–108. 18252251 10.1016/j.oraloncology.2008.01.008

[4] Miyazaki Y, Hara A, Kato K, Oyama T, Yamada Y, et al (2008) The effect of hypoxic microenvironment on matrix metalloproteinase expression in xenografts of human oral squamous cell carcinoma. Int J Oncol 32: 145–151. 18097553

[5] Bussink J, Kaanders JH, van der Kogel AJ (2003) Tumor hypoxia at the micro-regional level: clinical relevance and predictive value of exogenous and endogenous hypoxic cell markers. Radiother Oncol 67: 3–15. 12758235 10.1016/s0167-8140(03)00011-2

[6] Miyoshi A, Kitajima Y, Ide T, Ohtaka K, Nagasawa H, et al (2006) Hypoxia accelerates cancer invasion of hepatoma cells by upregulating MMP expression in an HIF-1-alpha-independent manner. Int J Oncol 29: 1533–1539. 17088993 10.3892/ijo.29.6.1533

[7] Munoz-Najar UM, Neurath KM, Vumbaca F, Claffey KP (2006) Hypoxia stimulates breast carcinoma cell invasion through MT1-MMP and MMP-2 activation. Oncogene 25: 2379–2392. 16369494 10.1038/sj.onc.1209273

[8] Guzy RD, Hoyos B, Robin E, Chen H, Liu L, et al (2005) Mitochondrial complex III is required for hypoxia-induced ROS production and cellular oxygen sensing. Cell Metab 1: 401–408. 16054089 10.1016/j.cmet.2005.05.001

[9] Kim YJ, Lee WS, Ip C, Chae HZ, Park EM, et al (2006) Prx1 suppressed radiation-induced c-Jun NH2 terminal kinase signaling in lung cancer cells through interaction with the glutathione S-transferase Pi/c-Jun NH2-terminal kinase complex. Cancer Res 66: 7136–7142. 16849559 10.1158/0008-5472.CAN-05-4446

[10] Neumann CA, Krause DS, Carman CV, Das S, Dubey DP, et al (2003) Essential role for the peroxiredoxin Prdx1 in erythrocyte antioxidant defence and tumoursuppression. Nature 424: 561–565. 12891360 10.1038/nature01819

[11] Yanagawa T, Omura K, Harada H, Ishii T, Uwayama J, et al (2005) Peroxiredoxin I expression in tongue squamous cell carcinomas as involved in tumor recurrence. Int J Oral Maxillofac Surg 34: 915–920. 15955662 10.1016/j.ijom.2005.04.015

[12] Yanagawa T, Iwasa S, Ishii T, Tabuchi K, Yusa H, et al (2000) Peroxiredoxin1 expression in oral cancer: a potential new tumor marker. Cancer Lett 156: 27–35. 10840156 10.1016/s0304-3835(00)00434-1

[13] Gandini NA, Fermento ME, Salomón DG, Blasco J, Patel V, et al (2012) Nuclear localization of heme oxygenase-1 is associated with tumor progression of head and neck squamous cell carcinomas. Exp Mol Pathol 93: 237–245. 22580187 10.1016/j.yexmp.2012.05.001

[14] Nakayama H, Ikebe T, Beppu M, Shirasuna K (2001) High expression levels of nuclear factor kappaB, IkappaB kinase alpha and Akt kinase in squamous cell carcinoma of the oral cavity. Cancer 92: 3037–3044. 11753981 10.1002/1097-0142(20011215)92:12<3037::aid-cncr10171>3.0.co;2-#

[15] Stanimirovic D, Zhang W, Howlett C, Lemieux P, Smith C (2001) Inflammatory gene transcription in human astrocytes exposed to hypoxia: roles of the nuclear factor-kappaB and autocrine stimulation. J Neuroimmunol 119: 365–376. 11585641 10.1016/s0165-5728(01)00402-7

[16] Jin DY, Chae HZ, Rhee SG, Jeang KT (1997) Regulatory role for a novel human thioredoxin peroxidase in NF-kappaB activation. J Biol Chem 272: 30952–30961. 9388242 10.1074/jbc.272.49.30952

[17] Hansen JM, Moriarty-Craige S, Jones DP (2007) Nuclear and cytoplasmic peroxiredoxin -1 differentially regulate NF-kappaB activities. Free Radic Biol Med 43: 282–288. 17603937 10.1016/j.freeradbiomed.2007.04.029 PMC2096473

[18] Bach FH (2005) Heme oxygenase-1: atherapeutic amplification funnel. FASEB J 19: 1216–1219. 16051687 10.1096/fj.04-3485cmt

[19] Otterbein LE, Choi AMK (2000) Heme oxygenase: Colors of defense against cellular stress. Am J Physiol Lung cell Mol Physiol 279: L1029–L1037. 11076792 10.1152/ajplung.2000.279.6.L1029

[20] Gandini NA, Fermento ME, Salomón DG, Blasco J, Patel V, et al (2012) Nuclear localization of heme oxygenase-1 is associated with tumor progression of head and neck squamous cell carcinomas. Exp Mol Pathol 93: 237–245. 22580187 10.1016/j.yexmp.2012.05.001

[21] Guo G, Bhat NR (2006) Hypoxia/reoxygenation differentially modulates NF-kappaB activation and iNOS expression in astrocytes and microglia. Antioxid Redox Signal 8: 911–918. 16771681 10.1089/ars.2006.8.911

[22] Mazza E, Thakkar-Varia S, Tozzi C, Neubauer JA (2001) Expression of hemeoxygenase in the oxygen-sensing regions of the rostral ventrolateral medulla. J Appl Physiol 91: 379–385. 11408455 10.1152/jappl.2001.91.1.379

[23] Nakaso K, Kitayama M, Mizuta E, Fukuda H, Ishii T, et al (2000) Co-induction of heme oxygenase-1 and peroxiredoxin I in astrocytes and microglia around hemorrhagic region in the rat brain. Neurosci Lett 293: 49–52. 11065135 10.1016/s0304-3940(00)01491-9

[24] Neumann CA, Fang Q (2007) Are peroxiredoxins tumor suppressors? Curr Opin Pharmacol 7: 375–380. 17616437 10.1016/j.coph.2007.04.007

[25] Dos Santos M, Mercante AM, Louro ID, Gonçalves AJ, de Carvalho MB, et al (2012) HIF1-Alpha Expression Predicts Survival of Patients with Squamous Cell Carcinoma of the Oral Cavity. PLoS One 7: e45228. 23028863 10.1371/journal.pone.0045228 PMC3445490

[26] Kim YJ, Ahn JY, Liang P, Ip C, Zhang Y, et al (2007) Human Prx1 gene is a target of Nrf2 and is up-regulated by hypoxia/reoxygenation: implication to tumor biology. Cancer Res 67: 546–554. 17234762 10.1158/0008-5472.CAN-06-2401

[27] Wang X, He S, Sun JM, Delcuve GP, Davie JR (2010) Selective association of peroxiredoxin 1 with genomic DNA and COX-2 upstream promoterelements in estrogen receptor negative breast cancer cells. Mol Biol Cell 21: 2987–2995. 20631257 10.1091/mbc.E10-02-0160 PMC2929992

[28] Ruiz-Ramos R, Lopez-Carrillo L, Rios-Perez AD, De Vizcaya-Ruíz A, Cebrian ME (2009) Sodium arsenite induces ROS generation, DNA oxidative damage, HO-1 and c Myc proteins, NF-kappaB activation and cell proliferation in human breast cancer MCF-7 cells. Mutat Res 674: 109–115. 18996220 10.1016/j.mrgentox.2008.09.021

[29] Liu PL, Tsai JR, Charles AL, Hwang JJ, Chou SH, et al (2010) Resveratrol inhibits human lung adenocarcinoma cell metastasis by suppressing heme oxygenase 1-mediated nuclear factor-kappaB pathway and subsequently downregulating expression of matrix metalloproteinases. Mol Nutr Food Res 54 Suppl 2 S196–204. 20461740 10.1002/mnfr.200900550

[30] Liu ZM, Chen GG, Ng EK, Leung WK, Sung JJ, et al (2004) Upregulation of heme oxygenase-1 and p21 confers resistance to apoptosis in human gastric cancer cells. Oncogene 23: 503–513. 14647439 10.1038/sj.onc.1207173

[31] Rushworth SA, Bowles KM, Raninga P, MacEwan DJ (2010) NF-kappaB-inhibited acute myeloid leukemia cells are rescued from apoptosis by heme oxygenase-1 induction. Cancer Res 70: 2973–2983. 20332229 10.1158/0008-5472.CAN-09-3407

[32] Allan ME, Storey KB (2012) Expression of NF-κB and downstream antioxidant genes in skeletal muscle of hibernating ground squirrels, Spermophilus tridecemlineatus. Cell Biochem Funct 30: 166–174. 22086848 10.1002/cbf.1832

[33] Lee HJ, Lee J, Min SK, Guo HY, Lee SK, et al (2008) Differential induction of heme oxygenase-1 against nicotine-induced cytotoxicity via the PI3K, MAPK, and NF-kappa B pathways in immortalized and malignant human oral keratinocytes. J Oral Pathol Med 37: 278–286. 18205746 10.1111/j.1600-0714.2007.00616.x

[34] Lavrovsky Y, Song CS, Chatterjee B, Roy AK (2000) Age-dependent increase of heme oxygenase-1 gene expression in the liver mediated by NF-kappaB. Mech Ageing Dev 114: 49–60. 10731581 10.1016/s0047-6374(00)00087-7

[35] Jeong SI, Choi BM, Jang SI (2010) Sulforaphane suppresses TARC/CCL17 and MDC/CCL22 expression through heme oxygenase-1 and NF-κB in human keratinocytes. Arch Pharm Res 33: 1867–1876. 21116791 10.1007/s12272-010-1120-6

[36] Riddell JR, Bshara W, Moser MT, Spernyak JA, Foster BA, et al (2011) Peroxiredoxin 1 controls prostate cancer growth through Toll-like receptor 4-dependent regulation of tumor vasculature. Cancer Res 71: 1637–1646. 21343392 10.1158/0008-5472.CAN-10-3674 PMC3076642

[37] Riddell JR, Maier P, Sass SN, Moser MT, Foster BA, et al (2012) Peroxiredoxin 1 stimulates endothelial cell expression of VEGF via TLR4 dependent activation of HIF-1α. PLoS One 7: e50394. 23185615 10.1371/journal.pone.0050394 PMC3503895

